# Progress in Medicine

**Published:** 1897-05-08

**Authors:** 


### PROGRESS IN MEDICINE.

### DISEASES OF THE DIGESTIVE ORGANS.

**Stomach.**—The diagnosis of dilatation has been exhaustively treated by W. Pepper and A. Stengel.[1] They consider that motor insufficiency or gastric atony is only a stage in this disease, and should be included under the name. On the other hand the size of the stomach, as well as its position, is so variable as to be of little value for the diagnosis of this affection. Cases are known where the capacity of one stomach has been shown to be five times as great as that of another person of the same height, and in some such instances there is no evidence of motor failure. The causes, then, of true dilatation are either those of pyloric obstruction or those of general atony, adhesions, and cicatrices. The patient is often emaciated, distension either below or to one side of the normal situation of the stomach may be present, and peristaltic waves may be seen crossing it. In a few cases we can actually feel the margin of the greater curvature or a pyloric growth. Succussion splashes in others at a time when the stomach ought to have passed on all its contents, especially if audible at a distance, are important symptoms. Percussion is unreliable, though Dehio's method of introducing water half-a-pint at a time, and percussing while the patient stands upright, is of some use in fixing the lower border, which in health never reaches the umbilicus. However, depression is due to other causes as well as dilatation. The authors rely far more on auscultatory percussion to determine the area of the stomach, and to confirm the results they employ inflation by air by a stomach tube and a simple Davidson's syringe. These two methods are quite satisfactory, but to establish the motor insufficiency they examine whether food is present in the viscus more than seven hours after a meal of meat, soup, and bread. Sour and ill-smelling vomits and a decrease of urine are sometimes aids to the diagnosis, especially with reference to the cases of megalogastria and gastroptosis mentioned above.

In the further diagnosis between malignant growths and other causes of the disease in a given case, they agree with various writers that there is no certain test for cancer, but that the presence of lactic acid and the absence of H.Cl., the existence of a tumour, and the occurrence of the disease in persons over 40 where there has been previously good digestion, are important evidence in favour of malignant growths.

Gasteroptosis is shown by Biol[2] to be very common in persons with deformed chests, or emphysema, and in many there are no gastric symptoms at all. Indeed, he lays down that it is not to be regarded as an ordinary cause of stomach troubles, though it may increase existing ones, or occasionally produce pain and hyperacidity, especially in women.

Diffuse or infiltrating cancer of the stomach is a rare affection, and may cause no dilatation. Douglas Powell[3] discusses a case of this disease in which an apparent tumour was felt to contract under the hand like a uterus. The patient vomited a little green fluid without H.Cl., and suffered from a little pain in the epigastrium, but the affection was almost impossible to diagnose. Schwyzer[4] reports an instance of congenital hypertrophy and constriction of the pylorus in an infant of three months, who was greatly emaciated, and had suffered from vomiting and occasional diarrhœa. No bile or fæcal matter was ever found in the vomit, such as occurs in obstruction of the bowels, from which this disorder has to be diagnosed. Pitt and others have reported a few similar cases, and the stenosis usually seems to depend on a hypertrophy of the circular fibres.

We have recently referred to two cases of phlegmonous gastritis, and now two more are reported from Russia, one of which recovered. Chanutin[5] summarises the symptoms as follows: Rigors, pyrexia, sweats, rapid pulse, vomiting of pus and bile, pain of a burning character, tenderness, and at times a tumour. In the absence of some of these the diagnosis is impossible. The solitary recovery above mentioned took place under merely expectant treatment, ice, morphia, and nutrient enemata.

Max Einhorn[6] discusses the production of constipation and of diarrhœa by gastric diseases. He opposes the view of Oppler that chronic diarrhœa is caused by achylia and constipation only by hyperchlorydria. This is, no doubt, the most frequent case, but the conditions may be reversed. Thus he quotes instances of hyperchlorydria accompanied with either state in which a cure was obtained by diet and half a drachm of bicarbonate of soda two hours after the three chief meals; also of achylia where a vegetable diet and other remedies relieved either condition. As an upholder of electrical treatment he combats the statement of Meltzer that faradisation cannot produce contractions in the stomach and intestines, and that the gastric mucosa opposes an extremely strong resistance to the passage of the current.[7] He shows by direct experiments on animals that contractions are easily produced by intragastric faradism or galvanism, and found the resistance to the passage of a galvanic current from within the stomach of a man to the epigastrium to be only 6,800 ohms, while it was 22,000 from the back to the front of his body. Murdoch[8] condemns the indiscriminate use of the stomach tube. He restricts its employment to the following: (1) For diagnostic purposes; (2) for cases of poisoning; (3) for stagnation of food in the stomach, for temporary relief of pain with other remedies; (4) for an excess of mucus in chronic gastritis, and sometimes in achylia; (5) for achylia, even where there is no such excess. In hæmorrhage, ulcer, aneurism, or great weakness it is contra-indicated. Benedict,[9] in reviewing the tests for acidity, believes that the reason of the colour change produced in the resorcin and sugar test with free mineral acid is that anilin red is formed from the aromatic radicle by an oxidising agent in the presence of acid. Thus he finds that carbolic acid and sugar give a red or green colour with strong or weak mineral acids. Lactic acid is produced, according to Stern,[10] in stagnation of the food with diminution of H.Cl. This occurs most often in cancer, but there are exceptions. Clinically Uffelman's test after Ewald's test breakfast is sufficient. The stomach may require first washing out, but it is unnecessary to exclude baked food or to use complicated tests to demonstrate its presence. In healthy stomachs the above test, even with Kelling's modification, shows no lactic acid. Manges[11] has written a valuable account of the digestive ferments, in which he shows the small value of pepsin and similar substances as usually given. Pepsin, for instance, is destroyed if dispensed with more than one per cent. of acid, or with antiseptics. It may be of temporary use in chronic catarrh, carcinoma, dilatation, atrophy of the mucosa, and the state resulting from phthisis and cardiac disease. If we are tempted to employ maltine or taka diastase for the faulty digestion of starches, they should be given with large doses of alkalies; the meal should include starches alone, and be well masticated. Sticker[12] gives strong proofs that the ammonia found in the stomach is derived from the saliva, and not produced by the peptic glands as Rosenheim taught. We may note here that methylene blue is of use in hyperacidity. One to three grains are given daily for a few days, and then intermitted.[13] The urine and fæces turn blue, but with no ill effects. It has been serviceable also in various forms of Bright's disease and gonorrhœa.

**Liver.**—Prevalent ideas on hepatic diseases are being rapidly modified. The formation and the surgical treatment of biliary calculi are subjects of much interest at the present time, whilst our knowledge of the production and course of cirrhosis has been almost revolutionised by recent researches. We shall refer later on to the mass of statistics accumulated by Foxwell, Kelynack, and Rolleston on the weight and size of cirrhotic livers and the results of the disease. Much brilliant work has been done according to Stanley[14] by the late Victor Hanot on the etiology of cirrhosis, in proving that it is often due to an auto intoxication. Bouchard had previously shown that an enlarged liver often follows dilatation of the stomach and other gastric disorders, as, indeed, we commonly see in children. Victor Hanot found that a form of cirrhosis often follows prolonged dyspepsia, and believed that microbial infections are a common cause, as well as alcohol. Boix, too, proved experimentally that butyric, lactic, and acetic acids could produce cirrhosis even more rapidly when alcohol is not given than when alcohol is added to the acid. Indeed, the administration of alcohol alone has usually failed to cause cirrhosis in animals, but if a previous defect of the liver exists alcohol affects it more easily. Do any infective processes prepare the way for cirrhosis? The bacillus septicus putridus, and pyocyaneus, as well as the infections of measles and scarlet fever, seem able to bring about cirrhosis, but the resisting power of individuals varies greatly. Indeed, Scagliosi affirms that excess of alcohol produces no effect on a perfectly healthy liver. The drunkard's liver, then, is probably due chiefly to infections from the duodenum and stomach, which are in such cases chronically inflamed and weakened by the alcohol. Levi notices that liver disease occasionally leads to coma and convulsions, either transitory or terminal, which may be mistaken for uræmia, and, indeed, a combined cirrhosis of kidneys and liver with rapidly fatal coma has been observed by him. T. S. Short[15] discusses the chief nervous affections of alcoholism. Among them are alcoholic coma; delirium tremens, with visual hallucinations, restlessness, fear, tremor, and failure of sleep and appetite; acute traumatic delirium occuring after wounds; mania *a potu* without tremor, but with raving and violence; multiple neuritis, with loss of knee-jerks, and tenderness over the calves and nerve trunks; chronic insanity, showing in different cases either physical, mental, or moral degeneration especially prominently. The statisticson cirrhosis compiled by Rolleston and Fenton[16] show the high rate of mortality from tuberculosis in this disease. The disease is commoner in males; the livers are on the average considerably increased in weight, especially in the alcoholic variety, which is produced both by spirits and malt liquors, and is commoner than that from other causes. The spleen is also enlarged in patients dying from cirrhosis. In young women a large liver and head symptoms are most frequent; in older women a small liver and ascites is the rule. For comparison with these 114 London cases, Kelynack[17] reports 121 from Manchester, and finds that 12 per cent. die from tuberculosis, and 50 per cent. from the cirrhosis itself. He does not think it possible to classify his cases as alcoholic and non-alcoholic, and lays down that the disease is commonest in males, and that the livers weigh on the average 53 or 54 ounces, being diminished in size in 61½ per cent. The spleen is generally enlarged. Renal cirrhosis is combined with the disease in 18½ per cent. of cases. Foxwell[18] states that in 202 private patients the liver was enlarged in 105; in only seven (and those non-alcoholic ones) did it seem smaller than normal. However, it may be replied that these were unverified by post-mortems; and Kelynack insists on the difference between the size of the liver in life and after death. Foxwell finally concludes upon other evidence that alcoholic cirrhosis is accompanied by enlargement, not only from hyperæmia, but also from new growth, and points out that the recognition of this fact aids the diagnosis of the disease.

Krawkow[19] has experimentally produced cirrhosis by introducing microbes into the alimentary canal, and therefore suggests that it may sometimes be due in human beings to the absorption of putrid matter from the intestines. By injection of the same microbic poisons into the flesh instead of into the intestines amyloid degeneration was produced. An instance of the rare disease hypertrophic pigmentary alcoholic cirrhosis is recorded by Letulle.[20] The pigment contained free iron, and could be found in the connective tissue cells generally over the body, but the spleen and skin were almost free. Another rare disease is reported by Adami,[21] that of "foaming liver." Perforative appendicitis led to the infection of the organ with the bacillus aërogenes capsulatus. It was found to be emphysematous, crackling on pressure, and when a lighted match was held near the cut surface, and pressure was exerted, the gas ignited in a series of small explosions. A similar case was noted also in Montreal about the same time by Jamieson. A case of jaundice of 14 months' duration from impaction of a gallstone in the common duct is discussed by E. M. Brocklebank.[22] The patient suffered from periodic pain, rigors, and vomiting, but there was no distension of the gall-bladder. All this pointed to an obstruction in the common duct by a calculus. The patient was still living in fair health when last heard of. Treatment consisted of Sapo durus, gr. xv. in pills, and ether in capsules ♏ xv. daily, with saline aperients. Hæmorrhage from the gums was noticed, and this, in the view of many surgeons, is a counter-indication to operation, as indicating a more or less uncontrollable bleeding from the gall-bladder, if opened. Another point of interest which the author dwells upon is the previous occurrence of typhoid in this patient. Since the discovery that this disease sometimes causes cholecystitis, and eventually gall-stones, the bacilli have been searched for and found during the fever in an active state in the gall-bladder rather frequently. Moreover, they survive there long after the fever is over, and may set up ulceration and even perforation. Thus the jaundice of this patient may be due to the effects of her fever seven years earlier.

B. S. Talmey[23] gives the history of a case of jaundice lasting 20 years, with frequent attacks of pain and rigors. A calculus was at last expelled while taking a course of calomel. The stone was egg-shaped, 3½ centimetres in length and 6½ in circumference. He accepts Naunyn's theory that the formation of gall-stones is due to stagnation of bile, which damages the epithelium, either directly or by a secondary infection producing a catarrh. The patient in question suffered from intermittent attacks of fever, which led to a diagnosis of abscess of the liver, though the symptom is by no means uncommon in calculus without abscess. Talmey lays stress on the value of a mixed diet, and the danger of morphia actually hindering the expulsion of a calculus. He advises the use of calomel, and if pain is severe gives half-drachm doses of ether.

I. Adler[24] reviews the diagnosis of gall-stones when jaundice is absent. They may exist without jaundice in the gall-bladder or cystic duct, or even in the hepatic or the common duct, if these latter are not entirely occluded. The most frequent symptom is pain either in the typical situation or in the pit of the stomach, but sometimes elsewhere, as in the ileo-cæcal region. This pain may be of varied character, often repeated, and is often accompanied by tenderness if the lower edge of the liver is struck by the hand. The gall-bladder may or may not be distended and palpable. Sometimes there is pyrexia, intermittent and gradually increasing, while the urine may show bilirubin. Difficult as diagnosis may be, if the attacks of pain become frequent and the calculi are not evacuated, if the gall-bladder is distended, and the temperature gradually rises, then he advises that surgical interference should be undertaken without waiting for possible spontaneous resolution. It is important, however, as A. McPhedran[25] remarks, to remember that tongue-like accessory lobes of the liver not infrequently simulate distended gall-bladders. Of these he records seven instances. The condition may be purely developmental, and unconnected with any disease. The difficulty of diagnosing malignant disease of the gall-bladder from gall-stone obstruction is discussed by Rolleston,[26] who points out that, as one disease so frequently follows the other, it is often impossible to say where malignant disease commenced. The progressive enlargement of the gall-bladder, especially if nodulated, cachexia, and secondary growths are important symptoms. Pyloric and pancreatic tumours may closely simulate those of the gall-bladder. Jaundice in malignant disease often reaches a greater degree of intensity than in mere obstruction but the diagnosis is beset with many difficulties. Rolleston raises the interesting question—How is it that the other "annexe" of the intestinal canal, the vermiform appendix, is never attacked by primary cancer, while the gall-bladder is so frequently subject to it? Is it owing to the highly developed state of the latter, and the atrophied one of the former? A case of primary sarcoma of the liver is reported by Byron Bramwell,[27] with references to the entire literature of the subject. Only twenty-five instances are recorded, many of which are doubtful. Bramwell's own case was at first taken for a hepatic abscess and aspirated.

[1] Amer. Jour. Med. Sciences, Jan. [2] Berlin Klin. Woch., Dec. 14. [3] Practitioner, Feb. [4] N. York Med, Jour., Nov. 21. [5] Amer. Jour. Med. Sciences, Feb. [6] Med. Record, Feb. 27. [7] New York Med. Jour., Dec. 12. [8] N. Y. Med. Jour., Jan. 16. [9] Amer. M. S. Bulletin, Dec. 26. [10] Amer. J. Med Sciences, Feb. [11] Amer. M. S. Bulletin, Dec. 12. [12] Amer. Jour. Med. Science, Feb. [13] Lancet, Jan. 30. [14] Birmingh. Med. Rev., Feb. [15] Birmingh. Med. Rev., March. [16] Birmingh. Med. Rev., Oct. [17] Birmingh. Med. Rev., March. [18] A. Foxwell, "Enlarged Cirrhotic Liver." [19] Brit. Med. Jour., Nov. 14. [20] Brit. Med. Jour., March 6. [21] Med. Chron., Nov. [22] Brit. Med. Journ. [23] New York Med. Jour. [24] New York Med. Jour., Feb. 27. [25] Practitioner, Dec. [26] Clinical Jour. Ap. 7. [27] Lancet, Jan. 16.

